# Altered expression patterns of lipid metabolism genes in an animal model of HCV core-related, nonobese, modest hepatic steatosis

**DOI:** 10.1186/1471-2164-9-109

**Published:** 2008-02-29

**Authors:** Ming-Ling Chang, Chau-Ting Yeh, Jeng-Chang Chen, Chau-Chun Huang, Shi-Ming Lin, I-Shyan Sheen, Dar-In Tai, Chia-Ming Chu, Wei-Pin Lin, Ming-Yu Chang, Chun-Kai Liang, Cheng-Tang Chiu, Deng-Yn Lin

**Affiliations:** 1 Liver Research Center and Department of Hepatogastroenterology, Chang Gung Memorial Hospital, No 5, Fu Hsing Street, Kuei Shan, Taoyuan, Taiwan; Chang Gung University, College of Medicine, Taoyuan, Taiwan; 2 Department of Surgery, Chang Gung Memorial Hospital, Taoyuan, Taiwan; 3 Division of Endocrinology & Metabolism, Department of Internal Medicine, Chang Gung Memorial Hospital, Taipei, Taiwan; 4 Division of Pediatric Critical Care and Emergency Medicine, Chang Gung Memorial Hospital, Taoyuan, Taiwan

## Abstract

**Background:**

Because the gene expression patterns of nonobese hepatic steatosis in affected patients remain unclear, we sought to explore these patterns using an animal model of nonobese hepatic steatosis.

**Methods:**

We developed mice that conditionally express the hepatitis C virus (HCV) core protein regulated by the tetracycline transactivator (tTA). Microarray analyses and reverse-transcription polymerase chain reaction were performed using liver samples of both the double transgenic mice (DTM), which express both the HCV core and tTA, and single transgenic mice (STM), which express tTA alone, at 2 months of age. Functional categories of genes with altered expression were classified using gene ontology programs. Serum glucose, lipid levels, and systemic blood pressure were also measured.

**Results:**

Approximately 20–30% of hepatocytes from the DTM were steatotic. No significant differences were observed in the serum glucose, lipid content, or blood pressure levels between the DTM and STM. Gene expression analyses revealed Sterol-regulatory element-binding protein (SREBP) pathway activation and dysregulation of the following genes involved in lipid metabolism: 3-hydroxy-3-methylglutaryl-coenzyme A synthase 1, Apolipoprotein AII, Apolipoprotein CI, acyl-CoA thioesterase I, and fatty acid binding protein 1; in mitochondrial function: solute carrier family 25 member 25 and cytochrome *c *oxidase subunit II; in immune reaction: complement component 3, lymphocyte antigen 6 complex, locus A, lymphocyte antigen 6 complex, locus C, lymphocyte antigen 6 complex, locus D, and lymphocyte antigen 6 complex, locus E.

**Conclusion:**

Some genes of lipid metabolism, mitochondrial function, and immune reaction and the SREBP pathway are involved in HCV core-related, nonobese, modest hepatic steatosis.

## Background

Nonalcoholic fatty liver disease (NAFLD) can be a severe, progressive liver disease leading to the development of cirrhosis [1]. Obesity and type 2 diabetes are well-known risk factors for the development of NAFLD. However, NAFLD is not rare in nonobese adults. The result of examining over 700 nonobese individuals older than 30 years with NAFLD who participated in medical examinations shows that NAFLD can be considered an early predictor of metabolic disorders for the normal-weight population [2]. Nevertheless, the basis for nonobese hepatic steatosis remains uncertain, particularly for those who lack any metabolic syndromes. Several experimental animal models for nonobese NAFLD have been proposed. Among them, cholesterol-fed rabbits share several physiopathological features of NAFLD, like hyperlipidemia, but are devoid of insulin resistance or obesity [3]; while overexpression of SREBP-1a in spontaneously hypertensive rat models were nonobese animals with hypertension, hepatic steatosis, and the metabolic syndrome [4]. All the above models are associated with metabolic syndrome in various degrees. Whether the basis of the "uncomplicated" nonobese hepatic steatosis is similar to hepatic steatosis complicated by obesity and/or metabolic syndrome remains unresolved. Hepatic steatosis is present in almost 50% of patients infected by hepatitis C virus (HCV), which therefore suggests it is an important contributor to NAFLD [5]. We aimed to study the gene expression involved in lipid metabolism of an animal model of nonobese hepatic steatosis free from metabolic syndrome based on the conditional HCV core transgenic mice developed from our previous work. Liver from the 2 month old conditional HCV core transgenic mice on chow without doxycycline (dox) showed that the severity of hepatic steatosis correlated with HCV core expression, peaked at the age of 2 months but diminished gradually [6].

## Results

### Fatty liver evaluation

**Figure 1:**
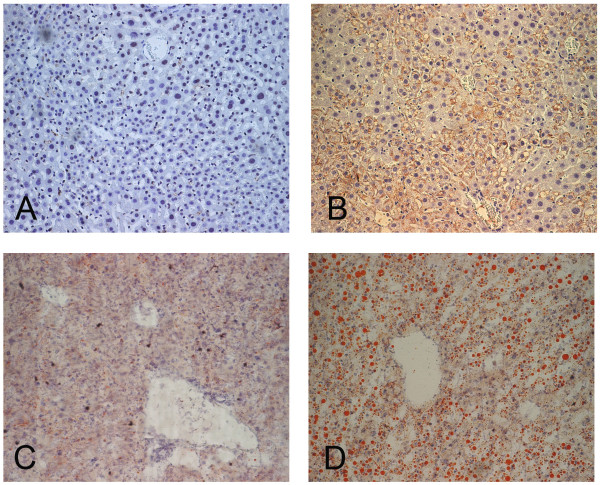
**Immunostaining of HCV core protein in STM (A, 20×) and DTM (B, 20×) livers fixed in 4% PFA, and the corresponding Oil Red O stain for fat in the livers of the frozen section (C, 20×; D, 20×).** The livers were from the mice at the age of 2 months old.

**Figure 2:**
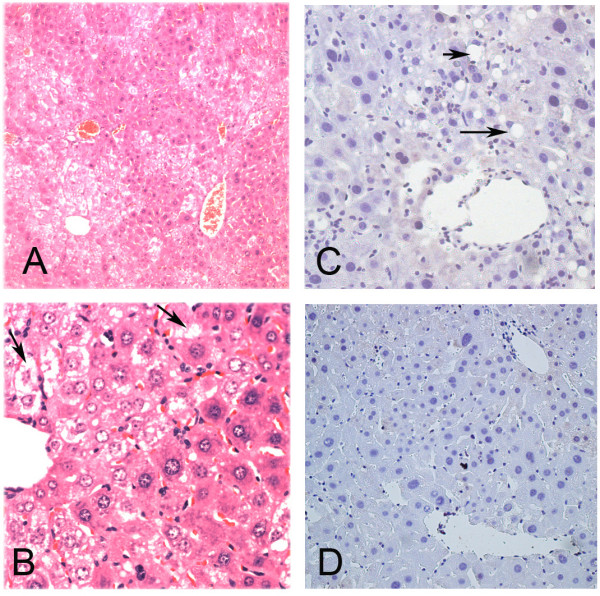
**H and E stain of DTM livers at 2 months (A, 20×; B, 40×); 4 months (C, 40×); and 6 mo of age (D, 40×).** The whitish areas (fatty area) intermixed with reddish areas was observed in the low power field (A). The characteristic microvesicular steatosis is denoted by arrows under high power field (B).

For the double transgenic mice (DTM), which express both the HCV core and tetracycline transactivator (tTA), oil red stain documented the existence of hepatic lipid vesicles that were compatible with HCV core protein in the parallel sections (Figure 1B and 1D). The severity of hepatic steatosis correlated with HCV core expression, and peaked at the age of 2 months, when the hepatic steatosis was microvesicular (Figure 2A and 2B, arrows). As the mouse aged, the microvesicular steatosis was replaced by macrovesicular steatosis (in a lesser degree, Figure 2C, arrows) and dimished gradually (Figure 2D). The proportions of steatotic hepatocytes were approximately 20–30% in the liver of 2 month old mice on chow without dox, which were examined under low power field for H and E stain (Figure 2A).

### Serum glucose, lipid, insulin and homeostatic model assessment for insulin resistance (HOMA-IR) evaluation

No significant differences in the serum glucose, lipid, insulin, or HOMA-IR levels between the DTM and single transgenic mice (STM), which express tTA alone, were noted. No definitive abnormal levels were found in any of the mice.

### Systemic blood pressure (SBP) measurement

There was no significant difference in the SBP level between the DTM and STM. No definitive abnormal levels were found in any of the mice.

### Microarray analyses

A total of 20871 genes were evaluated. Of these, 97% remained after filtering for missing data and/or low expression levels. Significance Analysis of Microarrays (SAM) revealed no sex bias in gene expression from the microarray data. For all of the 6 pairs, there were 28 genes that had M values (ie, log 2 Cy5/Cy3) ≥ 1, and 26 genes that had M values ≤ -1. Among the 28 up-regulated genes, those involved in lipid metabolism were as follows: serum amyloid A1 (*Saa1*), serum amyloid 3 (*Saa3*), 7-dehydrocholesterol reductase (*Dhcr7*) and 3-hydroxy-3-methylglutaryl-coenzyme A synthase 1 (*HMGCS1*). Among the 26 down-regulated genes, those involved in lipid metabolism were as follows: apolipprotein CI (*Apo CI*), apolipoprotein AII (*Apo AII*), Fatty acid binding protein 1, liver (*FABP1*), acyl-CoA thioesterase I (*Acot1*)*and *apolipoprotein E (*Apo E*) [see Additional file 1]. Gene functional analysis for the microarray data showed that only the acute-phase response had a significant P value and false discovery rate (FDR) (Table 1).

**Table 1:** Gene functional analysis for selected genes from microarray analyses

**Category**	**Genes in Category**	**Genes in category (%)**	**Genes in list in category**	**Genes in list in category (%)**	***P*-value**^†^	***FDR*_P**^‡^
Biological Process						
GO:6953: acute-phase response	19	0.189	3	17.65	0.0000	<.0001
GO:50896: response to stimulus	1910	19.01	8	47.06	0.0080	0.0637
Molecular Function						
GO:5215: transporter activity	1163	9.927	5	33.33	0.0123	0.1353

^†^Student's *t*-test.

^‡^Adjusted P-value for Benjamini and Hochberg method FDR.

### Quantitative real-time polymerase chain reaction (Q-RT-PCR)

Our Q-RT-PCR data were regarded as significant at M (ie, log2 Experiment/control for Q-RT-PCR) ≥ 0.6 or ≤ -0.6. For the four up-regulated genes involved in lipid metabolism, Q-RT-PCR of all the 6 pairs showed only *HMGCS1 *had M value higher than 0.6; for the five down-regulated genes involved in lipid metabolism, *Apo CI*, *Apo AII*, *Acot1 *and *FABPl *showed M values less than -0.6. The mean and standard deviation of M values of Q-RT-PCR for the various genes are listed in Table 2.

**Table 2:** List of the mean and standard deviation of M values for Q-RT-PCR for genes involved in lipid metabolism.

***Gene name***	*Saa1*	*Saa3*	*HMGCS1*	*Dhcr7*	*ApoE*	*ApoCI*	*ApoAII*	*FABP1*	*Acot1*
**Mean +/- SD**	0.35 +/- 0.67	0.67 +/- 1.13	2.58 +/- 1.01	0.78 +/- 1.52	0.02 +/- 0.49	-2.05 +/- 1.12	-1.98 +/- 0.58	-1.77 +/- 0.62	-2.12 +/- 1.20

### Pathway classification

ArrayXPath identified 5 out of 79 input elements in 9 out of 277 BioCarta pathways. The pathways are shown in Table 3. Among the 9 pathways, 7 pathways showed significance with both P-values and Q-values less than 0.05. The Sterol-regulatory element-binding protein (SREBP) pathway composed of *SREBF1*, *SREBF2*, *MBPTS1*, *SCAP*, *LDLR *and *HMGCS1 *is involved in lipid metabolism. *HMGCS1 *activation was identified in the current study.

**Table 3:** Activated pathway identified from ArrayXpath website.

**pathway**	**Identified node**	**p-value**	**q-value**
Classical Complement Pathway	2/11 (60)	0.0007	0.0036
Complement Pathway	2/16 (30)	0.0015	0.0038
SREBP control of lipid synthesis	1/6 (27)	0.0236	0.0319
Alternative Complement Pathway	1/8 (43)	0.0314	0.0319
Fibrinolysis Pathway	1/11 (14)	0.0430	0.0319
Double Stranded RNA Induced Gene Expression	1/10 (14)	0.0391	0.0319
Extrinsic Prothrombin Activation Pathway	1/13 (24)	0.0506	0.0329
Lectin Induced Complement Pathway	1/10 (52)	0.0391	0.0339
Intrinsic Prothrombin Activation Pathway	1/23 (36)	0.0881	0.051

Identified node: matched nodes/unique nodes in pathway (redundant nodes in pathway).

## Discussion

We developed an animal model of nonobese hepatic steatosis based on mice conditionally expressing the HCV core protein. Doxycycline (Dox) chow, which contained an analog of tetracycline, was used to regulate the transgene expression. Dox is regarded as a steatogenic drug by means of inhibiting mitochondrial fatty acid beta-oxidation [7]. To avoid the bias of drug-related hepatic steatosis, we prescribed both the DTM and STM with the same course of Dox administration. We also ensured that the hepatic steatosis was due to expression of the HCV core transgene by comparing the liver biopsies from the DTM and STM. The former did show significant hepatic steatosis compared to the latter.

HCV core protein is known to play crucial roles in host cell lipid metabolism both *in vitro *and *in vivo *[8-13]. Gene expression profiles have been reported after HCV core expression in primary hepatocytes [14], hepatoma cell lines, [15] and yeast cells [16]. However, the data obtained for hepatic steatosis *in vitro *might not mirror that obtained *in vivo*. The expression of HCV core leads to progressive hepatic steatosis in several lines of constitutive transgenic mice [17]. Hepatocellular carcinoma (HCC) also results in some of these older mice due to oxidative stress [18]. Thus, in those constitutive transgenic mice, hepatic steatosis is a relay of HCC and may be the sequel of carcinogenic gene expression. In the current model, HCV core expression was robust and paralleled the degree of hepatic steatosis. Both core expression and hepatic steatosis peaked at 2 months but diminished gradually afterward. We did not find HCC in these mice, regardless of their age, partly due to HCV core-related, augmented hepatocellular apoptosis [6]. Therefore, because we established the exact timing of peak hepatic steatosis emergence (2 months) and these mice did not develop HCC, we propose that this is a valid model to study the basis of hepatic steatosis without bias. None of the transgenic mice showed definitively abnormal serum glucose, lipid, insulin, or HOMA-IR levels, and the blood pressure remained normal. The gene expression profiles of nondiabetic and diabetic ob/ob mice reported by Lan et al. suggest that increased hepatic lipogenic capacity protects the ob/ob mice from the development of type 2 diabetes [19]. Cumulatively, these observations indicate a lack of metabolic syndrome in these conditional HCV core mice that may have resulted from an only modest level of steatosis, which protects these mice from diabetes but is not sufficiently vigorous to cause metabolic syndrome.

Gene functional analysis of the microarray data showed that only the genes for an acute-phase response had significant P values and FDR (Table 1). These results are compatible with the observation that HCV core expression leads to augmented oxidative stress and hepatocellular apoptosis, but not subsequent hepatic inflammation and fibrosis in DTM [6]. However, several genes involved in mitochondrial function (*Slc25a25 *and *COX2*), the immune reaction (*C3*, *Ly6a*, *Ly6c*, *Ly6d*, and *Ly6e*), coagulation (*Fgb*), the cell cycle (*G0s2*), cell differentiation (*Onecut1 and Gadd45g*), cell proliferation (*Ifitm3*), apoptosis (*Bbc3*), angiogenesis (*Anxa2*), and cytochrome P450 function (*Cyp4a14*) were also regulated in the livers of DTM (see Additional file 1). At the basis of gene expression, the data did not conclusively explain why those mice did not develop HCC. For example, the downregulation of *Onecut1 *and upregulation of *Ifitm3 *might inhibit cell proliferation [20,21], whereas the downregulation of *GADD45G *might enhance tumor cell growth [22]. Several attempts were performed to approach the gene expression for human NAFLD. A study on microarray analyses including 62 human liver samples showed that mitochondrial alterations play a major role in the development of steatosis per se and the activation of inflammatory pathways is present at a very early stage of steatosis, even if no morphological sign of inflammation is observed [23]. In addition, the analyses of global hepatic gene expression in histologically progressive nonalcoholic steatohepatitis revealed down-regulated genes for maintaining mitochondrial function and up-regulated genes of C3 and hepatocyte-derived fibrinogen-related protein [24]. Therefore, mitochondrial function alteration and C3-implicated immune reaction/inflammation (complementary pathway activation is documented in Table 3) are likely to contribute at least partly to the hepatic steatosis in the current transgenic mice based on human NAFLD gene expression studies. Cyp4a enzymes were identified as initiators of oxidative stress in the liver of mice fed a methionine- and choline-deficient (MCD) diet, while the role of Cyp4a14 in hepatic steatosis demands further clarification [25].

We focused our analysis of the microarray data, Q-RT-PCR, and the gene nodal classifications on lipid metabolism. The livers of DTM showed one up-regulated gene, *HMGCS1*; four down-regulated genes, *Apo CI*, *Apo AII, Acot1*, and *FABPl*; and one activated pathway, SREBP-mediated control of lipid synthesis. HCV core expression decreased hepatic lipoprotein secretion and caused steatosis as shown using a line of constitutive HCV core transgenic mice [26]. By transfecting cell lines with HCV core plasmids, Barba et al. showed the colocalization of HCV core protein and Apo AII [8]. The direct binding of HCV core with Apo AII was also documented [27]. Moreover, hepatic human *Apo AII *expression in HCV core protein/*Apo AII*^-/- ^transgenic mice diminished intrahepatic core protein accumulation, whereas the converse scenario is highly possible, although uncertain, based on our result [13]. Little data directly address the relationships between HCV core-related steatosis and *Apo CI*. The infectivity of HCV pseudotyped retroviral particles is enhanced by *Apo CI *[28]. This observation suggests that a correlation may exist between *Apo CI *and the HCV core because the HCV core protein plays an important role in infectivity.

Acot1 is an enzyme that hydrolyzes long-chain acyl-CoAs of C(12)-C(20)-CoA in chain length to the free fatty acid and coenzyme A (CoA) [29]. The potency of Acot1 may serve to modulate intracellular concentrations of acyl-CoAs, free fatty acids, and CoA to affect various cellular functions, including lipid metabolism [30]. The downregulation of Acot1 likely leads to increased Acyl-CoA and subsequent beta oxidation, which might counter-regulate hepatic steatosis secondary to HCV core expression [31].

In regard to the SREBP pathway, several *in vivo *systems have documented its association with HCV or the HCV core protein. During the early stages of acute HCV infection, chimpanzees that develop either transient or sustained clearance of virus show activated genes involved in the SREBP pathway as determined by genome-wide transcriptional analyses [32]. HCV infection is also known to induce the proteolytic cleavage and phosphorylation of SREBPs via oxidative stress [33]. Furthermore, genes related to fatty acid biosynthesis and *SREBP-1c *promoter activity are up-regulated by the HCV core protein in cell lines and constitutive HCV core transgenic mice livers in a *PA28gamma*-dependent manner [34]. Our results demonstrate that aside from SREBPs themselves, HMGCS1, the crucial enzyme for the SREBP pathway, also plays some role in HCV core related hepatic steatosis. Whether HMGCS1 activation is specific to HCV core expression or is subsequent to hepatic steatosis demands further clarification.

To our knowledge, *FABP1 *has not been reported as being responsible for HCV core-related steatosis. The primary role of all the *FABP *family members is the regulation of fatty acid uptake and intracellular transport [35]. The *FABP1 *gene is turned on by long-chain fatty acids and nonmetabolized fatty acids in a physiologically relevant manner [36]. *FABP1 *and microsomal triglyceride transfer protein (MTP) are known to shunt fatty acids cooperatively into *de novo *synthesized glycerolipids and transfer lipids into very low-density lipoprotein (VLDL), respectively, which act together to maintain hepatic lipid homeostasis [37]. It is likely that either the HCV core leads to hepatic steatosis by means of modulating *FABPl *gene expression to inhibit MTP protein activity and VLDL secretion [38] or that downregulation of *FABPl *is a nonspecific phenomenon secondary to hepatic steatosis.

## Conclusion

We developed an animal model of nonobese hepatic steatosis with the transgenic expression of the HCV core protein. These mice were free of metabolic syndrome. The degree of hepatic steatosis paralleled the expression of the HCV core protein, and both core protein expression and steatosis diminished with time. Gene expression analysis of liver RNA from the transgenic mice showed that several genes involved in lipid transport, mitochondrial function, the immune reaction, and inflammation were either up- or down-regulated. The SREBP pathway was also activated as shown by the upregulation of the *HMGCS1 *gene. Our model lends itself to studying the gene expression patterns in nonobese hepatic steatosis, especially those associated with HCV infection.

## Methods

### Transgenic mice regeneration

Mice conditionally expressing HCV core gene were generated as described [6]. Briefly, the HCV core gene sequence was isolated by reverse-transcription RT-PCR from the plasma of a patient with chronic HCV genotype 1b. It was cloned into the pUGH16-3 vector, which contains the tetracycline response element [39]. Fertilized ova from FVB/N mice were injected with the construct, and several founder mice were obtained. These were crossed with a second transgenic line that is homozygous for the tTA, under the control of the liver activator protein (LAP) promoter, which is hepatocyte-specific [40]. The LAP-tTA mice were generously provided by J. M. Bishop and Rong Wang (University of California, San Francisco, CA). Unless otherwise indicated, mating pairs were maintained on dox-containing chow (doxycycline (200 mg)/chow (kg), Bio-serve, Frenchtown, NJ) to suppress the HCV core during development and through weaning (1 month old). At approximately 1 month of age, dox was withdrawn. The Animal Care and Use Committee at Chang Gung Memorial Medical Center approved the use of animals for this study.

### HCV core protein expression

Analyses of HCV core protein expression was performed by immunohistochemical staining as described previously for the DTM and STM [6].

### Fatty liver evaluation

Fat vesicles were identified by Oil Red O staining in frozen liver sections using a commercial kit (BioGenex, San Ramon, CA) according to the manufacturer's protocol. Hematoxylin and eosin (H and E) stains were also performed for the fatty liver grading.

### Serum glucose and lipid evaluation

Tail bleedings for biochemistry data evaluation were performed in 10 DTM (5 males and 5 females) and 10 STM (5 males and 5 females) at 2 months of age. The assays for glucose, uric acid, triglyceride, and cholesterol (Vitros DT60 II Chemistry System, Johnson & Johnson, Rochester, NY) were adopted for using tail blood according to the manufacturer's protocol after 12 hours of fasting.

### Insulin evaluation

To determine the serum insulin levels, ELISA bioassay kits for insulin (Crystal Chem Inc., Downers Grove, IL) were adopted according to the manufacturer's protocol.

### HOMA-IR

By using the formula: $\frac{\text{glucose in mg/dLX0}\text{.05551Xinsulin in UIU/mL}}{22.5}$

the HOMA-IR was calculated for the fasting mice serum.

### SBP

SBP was measured in conscious mice (10 DTM and 10 STM, respectively) by tail-cuff plethysmography (Model 179 blood pressure analyzer; Hugo Sachs Elektronik, Hugstetten, Germany) as described previously [41].

### Microarray analyses

Microarray analyses were used to obtain global gene expression profiles from the livers of three pairs of 2 month old female DTM versus 2 month old female STM; and three pairs of 2 month old male DTM versus 2 month old male STM. Since appropriate RNA pooling can provide equivalent power and improve the efficiency and cost-effectiveness of microarray experiments with a modest increase in the total number of subjects [42], pooled samples from three STM individuals were used as controls. Experimental procedures were carried out according to the manufacturer's protocols. Briefly, 0.5 μg of total RNA was amplified by a Fluorescent Linear Amplification Kit (Agilent Technologies, Santa Clara, CA) and labeled with Cy3-CTP or Cy5-CTP (CyDye, PerkinElmer, Waltham, MA) during the *in vitro *transcription process. RNA from DTM was labeled with Cy5 and RNA from STM was labeled with Cy3. Cy-labeled cRNA (2 μg) was fragmented to an average size of about 50–100 nucleotides by incubating with fragmentation buffer (Agilent Technologies) at 60°C for 30 minutes. Correspondingly, fragmented labeled cRNA was then pooled and hybridized to a mouse oligonucleotide microarray containing 20,871 unique mouse genes (Agilent Technologies) at 60°C for 17 h. After washing and drying by nitrogen gun blowing, microarrays were scanned with an Agilent microarray scanner (Agilent Technologies) at 535 nm for Cy3 and 625 nm for Cy5. Scanned images were analyzed by Feature Extraction Software 8.1 (Agilent Technologies). Image analysis and normalization software was used to quantify signal-to-background intensity for each feature, substantially normalizing the data by the rank-consistency-filtering LOWESS method [43]. Sex bias analysis was performed using Significance Analysis of Microarrays (SAM) [44], and P-values of <0.05 were considered as statistically significant.

### Q-RT-PCR

Q-RT-PCR was performed for the genes involved in lipid metabolism significantly up or down regulated in the microarray (log 2 Cy5/Cy3, ie, M ≥ 1 or ≤ -1) using the same RNA isolated for microarray analysis. To prepare a cDNA pool from each RNA sample, total RNA (5 μg) was reverse transcribed using MMLV reverse transcriptase (Promega, Madison, WI), and the resulting samples were diluted 40 times by volume with nuclease-free water. Each cDNA pool was stored at -20°C until further real-time PCR analysis. Real-time PCR reactions were performed on the Roche LightCycler Instrument 1.5 (Roche, Indianapolis, IN) using LightCycler^® ^FastStart DNA Master^PLUS ^SYBR Green I kit (Roche) according to manufacturer's protocol. The sequences of the primers for *Saa1*, *Saa3*, *HMGCS1*, *Dhcr7*, *Apo E*, *Apo CI*, *Apo AII*, *FABP1*, and *Acot1 *are shown in Table 4.

**Table 4:** Primers used for real-time PCR of genes involved in lipid metabolism.

**Gene**	**Primer sequence**
Saa1	F: 5'-GGAGACACCAGGATGAAGCTA-3'
	R: 5'-TAGGCTCGCCACATGTCC-3'
Saa3	F: 5'-TGCTCGGGGGAACTATGAT-3'
	R: 5'-ACAGCCTCTCTGGCATCACT-3'
HMGCS1	F: 5'-CCCCTTCACAAATGACCACAG-3'
	R: 5'-GACAGCTGATTCAGATTCGGC-3'
Dhcr7	F: 5'-TACCTAGGCTGGGGAGATTG-3'
	R: 5'-GGGTGGTACACCAAGTACAGG-3'
Apo E	F: 5'-CACGAGCGTCACTTCTTGG-3'
	R: 5'-CAGGAAAGGGTCCAGGTTCT-3'
Apo CI	F: 5'-CCTGATTGTGGTCGTAGCC-3'
	R: 5'-CCGGTATGCTCTCCAATGTT-3'
Apo AII	F: 5'-CCATCTGTAGCCTGGAAGGA-3'
	R: 5'-GTACTGAGTGAACAGGCTCTGC-3'
FABP1	F: 5'-CCATGACTGGGGAAAAAGTC-3'
	R: 5'-GCCTTTGAAAGTTGTCACCAT-3'
Acot1	F: 5'-CTGGCGCATGCAGGATC-3'
	R: 5'-CTGGCGCATGCAGGATC-3'

F, forward primer; R, reverse primer.

### Pathway classification

Only gene expression data with M values ≥ 1 or ≤ -1 were analyzed for potential pathways.

Potential pathways that were activated in the DTM were investigated using a web-based service [45], where Fisher's exact test and the FDR followed the Storey's scheme were applied to evaluate the statistical significance.

### Statistical analyses

Normalized microarray data were further filtered for missing genes and for genes with low expression levels. Considering multiple comparisons, the adjusted P-values for the Benjamini and Hochberg method FDR were also calculated for the selected genes, and an adjusted P-value of less than 0.05 was chosen for subsequent functional and pathway exploration. Analyses were accomplished using the SAS 8.0 statistical package (SAS Institute Inc., Cary, NC), and P < 0.05 was considered as statistically significant. Gene Ontology Generic GO slim in GeneSpring v.7.3.1 (Agilent Technologies) was used for the functional category classification (Student's *t*-test was used in GO).

## Authors' contributions

MLC, CTY, JCC, CCH, SML, ISS, DIT, CMC, WPL, MYC, CKL, CTC, and DYL had made substantial contributions to acquisition of data and analysis and interpretation of data. MLC had been involved in designing experiments and drafting the manuscript. CTY had revised the paper critically for important intellectual content. All the authors had given final approval of the version to be published.

## Supplementary Material

Additional file 1 List of differentially expressed genes potentially associated with pathogenesis of HCV core protein. Click here for file
