# Supplementary material for: Altered expression patterns of lipid metabolism genes in an animal model of HCV core-related, nonobese, modest hepatic steatosis
Source: BMC Genomics. 2008 Feb 29;9:109. doi: 10.1186/1471-2164-9-109 (PMC2287171; doi:10.1186/1471-2164-9-109)
Supplement: Additional file 1 — List of differentially expressed genes potentially associated with pathogenesis of HCV core protein. [file 1471-2164-9-109-S1.doc]

**Table.** List of differentially expressed genes potentially associated with pathogenesis of HCV core protein

| ***Function*** | ***Gene name*** | ***Description*** | ***Systemic name*** | ***M values of microarray*** |
| --- | --- | --- | --- | --- |
| [transport](http://www.ncbi.nlm.nih.gov/entrez/utils/fref.fcgi?http://amigo.geneontology.org/cgi-bin/amigo/go.cgi?view=details&depth=1&query=6810) ([pheromone binding](http://www.ncbi.nlm.nih.gov/entrez/utils/fref.fcgi?http://amigo.geneontology.org/cgi-bin/amigo/go.cgi?view=details&depth=1&query=5550)) | Mup1 | major urinary protein 1 | NM_031188 | -2.610+/-0.610 |
| [cell differentiation](http://www.ncbi.nlm.nih.gov/entrez/utils/fref.fcgi?http://amigo.geneontology.org/cgi-bin/amigo/go.cgi?view=details&depth=1&query=30154) ([DNA binding](http://www.ncbi.nlm.nih.gov/entrez/utils/fref.fcgi?http://amigo.geneontology.org/cgi-bin/amigo/go.cgi?view=details&depth=1&query=3677)) | Onecut1 | one cut domain, family member 1 | NM_008262 | -2.07+/-0.583 |
| [cell cycle](http://www.ncbi.nlm.nih.gov/entrez/utils/fref.fcgi?http://amigo.geneontology.org/cgi-bin/amigo/go.cgi?view=details&depth=1&query=7049) | G0s2 | G0/G1 switch gene 2 | NM_008059 | -2.044+/-0.656 |
| [heme biosynthetic process](http://www.ncbi.nlm.nih.gov/entrez/utils/fref.fcgi?http://amigo.geneontology.org/cgi-bin/amigo/go.cgi?view=details&depth=1&query=6783) ([5-aminolevulinate synthase activity](http://www.ncbi.nlm.nih.gov/entrez/utils/fref.fcgi?http://amigo.geneontology.org/cgi-bin/amigo/go.cgi?view=details&depth=1&query=3870)) | Alas1 | aminolevulinic acid synthase 1 | NM_020559 | -1.937+/-0.710 |
| [electron transport](http://www.ncbi.nlm.nih.gov/entrez/utils/fref.fcgi?http://amigo.geneontology.org/cgi-bin/amigo/go.cgi?view=details&depth=1&query=6118) (cytochrome P450, family 4, subfamily a, polypeptide 14: [alkane 1-monooxygenase activity](http://www.ncbi.nlm.nih.gov/entrez/utils/fref.fcgi?http://amigo.geneontology.org/cgi-bin/amigo/go.cgi?view=details&depth=1&query=18685)) | Cyp4a14 | cytochrome P450, family 4, subfamily a, polypeptide 14 | NM_007822 | -1.935+/-0.413 |
| [endopeptidase inhibitor activity](http://www.ncbi.nlm.nih.gov/entrez/utils/fref.fcgi?http://amigo.geneontology.org/cgi-bin/amigo/go.cgi?view=details&depth=1&query=4867) | Serpina3c | serine (or cysteine) peptidase inhibitor, clade A, member 3C | NM_008458 | -1.777+/-0.370 |
| [protein binding](http://www.ncbi.nlm.nih.gov/entrez/utils/fref.fcgi?http://amigo.geneontology.org/cgi-bin/amigo/go.cgi?view=details&depth=1&query=5515) (cell differentiation and apoptosis) | Gadd45g | growth arrest and DNA-damage-inducible 45 gamma | NM_011817 | -1.687+/-0.348 |
| [endopeptidase inhibitor activity](http://www.ncbi.nlm.nih.gov/entrez/utils/fref.fcgi?http://amigo.geneontology.org/cgi-bin/amigo/go.cgi?view=details&depth=1&query=4866) | Serpina1d | serine (or cysteine) peptidase inhibitor, clade A, member 1d | NM_009246 | -1.619+/-0.202 |
| [lipid transport](http://www.ncbi.nlm.nih.gov/entrez/utils/fref.fcgi?http://amigo.geneontology.org/cgi-bin/amigo/go.cgi?view=details&depth=1&query=6869) *([lipid transporter activity](http://www.ncbi.nlm.nih.gov/entrez/utils/fref.fcgi?http://amigo.geneontology.org/cgi-bin/amigo/go.cgi?view=details&depth=1&query=5319)) | Apoc1 | apolipoprotein C-I | NM_007469 | -1.593+/-0.120 |
| [fatty acid binding](http://www.ncbi.nlm.nih.gov/entrez/utils/fref.fcgi?http://amigo.geneontology.org/cgi-bin/amigo/go.cgi?view=details&depth=1&query=5504)* | Fabp1 | fatty acid binding protein 1, liver | NM_017399 | -1.588+/-0.213 |
| [transport](http://www.ncbi.nlm.nih.gov/entrez/utils/fref.fcgi?http://amigo.geneontology.org/cgi-bin/amigo/go.cgi?view=details&depth=1&query=6810) (mitochondrial carrier, phosphate carrier: [calcium ion binding](http://www.ncbi.nlm.nih.gov/entrez/utils/fref.fcgi?http://amigo.geneontology.org/cgi-bin/amigo/go.cgi?view=details&depth=1&query=5509)) | Slc25a25 | solute carrier family 25 (mitochondrial carrier, phosphate carrier), member 25 | NM_146118 | -1.563+/-0.447 |
| [carboxylesterase activity](http://www.ncbi.nlm.nih.gov/entrez/utils/fref.fcgi?http://amigo.geneontology.org/cgi-bin/amigo/go.cgi?view=details&depth=1&query=4091) | Es22 | esterase 22 | NM_133660 | -1.479+/-0.471 |
| [protein binding](http://www.ncbi.nlm.nih.gov/entrez/utils/fref.fcgi?http://amigo.geneontology.org/cgi-bin/amigo/go.cgi?view=details&depth=1&query=5515) (MAGUK p55 subfamily member 6: [guanylate kinase activity](http://www.ncbi.nlm.nih.gov/entrez/utils/fref.fcgi?http://amigo.geneontology.org/cgi-bin/amigo/go.cgi?view=details&depth=1&query=4385)) | Mpp6 | membrane protein, palmitoylated 6 | NM_019939 | -1.435+/-0.411 |
| [electron transport](http://www.ncbi.nlm.nih.gov/entrez/utils/fref.fcgi?http://amigo.geneontology.org/cgi-bin/amigo/go.cgi?view=details&depth=1&query=6118) ([cytochrome-c oxidase activity](http://www.ncbi.nlm.nih.gov/entrez/utils/fref.fcgi?http://amigo.geneontology.org/cgi-bin/amigo/go.cgi?view=details&depth=1&query=4129)) | COX2 | cytochrome c oxidase subunit II | AF378830 | -1.432+/-0.367 |
| [transcription regulator activity](http://www.ncbi.nlm.nih.gov/entrez/utils/fref.fcgi?http://amigo.geneontology.org/cgi-bin/amigo/go.cgi?view=details&depth=1&query=30528) | Id2 | inhibitor of DNA binding 2 | NM_010496 | -1.40+/-0.354 |
| [complement activation](http://www.ncbi.nlm.nih.gov/entrez/utils/fref.fcgi?http://amigo.geneontology.org/cgi-bin/amigo/go.cgi?view=details&depth=1&query=6956) | C3 | complement component 3 | NM_009778 | -1.374+/-0.184 |
| [lipid transporter activity](http://www.ncbi.nlm.nih.gov/entrez/utils/fref.fcgi?http://amigo.geneontology.org/cgi-bin/amigo/go.cgi?view=details&depth=1&query=5319)* | Apoa2 | apolipoprotein A II | NM_013474 | -1.333+/-0.116 |
| [lipid transporter activity](http://www.ncbi.nlm.nih.gov/entrez/utils/fref.fcgi?http://amigo.geneontology.org/cgi-bin/amigo/go.cgi?view=details&depth=1&query=5319)* | Apoe | apolipoprotein E | NM_009696 | -1.286+/-0.190 |
| [negative regulation of protein kinase activity](http://www.ncbi.nlm.nih.gov/entrez/utils/fref.fcgi?http://amigo.geneontology.org/cgi-bin/amigo/go.cgi?view=details&depth=1&query=6469) | Dnajc3 | DnaJ (Hsp40) homolog, subfamily C, member 3 | NM_008929 | -1.243+/-0.217 |
| [protein binding](http://www.ncbi.nlm.nih.gov/entrez/utils/fref.fcgi?http://amigo.geneontology.org/cgi-bin/amigo/go.cgi?view=details&depth=1&query=5515) ([ubiquitin cycle](http://www.ncbi.nlm.nih.gov/entrez/utils/fref.fcgi?http://amigo.geneontology.org/cgi-bin/amigo/go.cgi?view=details&depth=1&query=6512)) | Spop | speckle-type POZ protein | NM_025287 | -1.219+/-0.216 |
| [blood coagulation](http://www.ncbi.nlm.nih.gov/entrez/utils/fref.fcgi?http://amigo.geneontology.org/cgi-bin/amigo/go.cgi?view=details&depth=1&query=7596) | Fgb | fibrinogen beta chain | NM_181849 | -1.203+/-0.418 |
| hypothetical protein | BC071254 |  | BC071254 | -1.20+/-0.385 |
| [transport](http://www.ncbi.nlm.nih.gov/entrez/utils/fref.fcgi?http://amigo.geneontology.org/cgi-bin/amigo/go.cgi?view=details&depth=1&query=6810) ([retinoid binding](http://www.ncbi.nlm.nih.gov/entrez/utils/fref.fcgi?http://amigo.geneontology.org/cgi-bin/amigo/go.cgi?view=details&depth=1&query=5501)) | Rbp4 | retinol binding protein 4, plasma | NM_011255 | -1.165+/-0.250 |
| Lipid metabolism * ([acyl-CoA thioesterase I activity](http://www.ncbi.nlm.nih.gov/entrez/utils/fref.fcgi?http://amigo.geneontology.org/cgi-bin/amigo/go.cgi?view=details&depth=1&query=16292)) | Acot1 | acyl-CoA thioesterase 1 | NM_012006 | -1.125+/-0.334 |
| hypothetical protein | 4930461P20Rik |  | XM_127961 | -1.09+/-0.214 |
| [regulation of transcription](http://www.ncbi.nlm.nih.gov/entrez/utils/fref.fcgi?http://amigo.geneontology.org/cgi-bin/amigo/go.cgi?view=details&depth=1&query=45449) | Klf10 | Kruppel-like factor 10 | NM_013692 | -1.052+/-0.242 |
| Defense response (glycosylphosphatidylinositol binding) | Ly6a | lymphocyte antigen 6 complex, locus A | NM_010738 | 3.5175+/-0.175 |
| Defense response (glycosylphosphatidylinositol binding) | Ly6d | lymphocyte antigen 6 complex, locus D | NM_010742 | 3.281+/-1.097 |
| [integral to membrane](http://www.ncbi.nlm.nih.gov/entrez/utils/fref.fcgi?http://amigo.geneontology.org/cgi-bin/amigo/go.cgi?view=details&depth=1&query=16021) | 1810023F06Rik | RIKEN cDNA 1810023F06 gene | NM_145449 | 2.652+/-0.396 |
| [ubiquitin cycle](http://www.ncbi.nlm.nih.gov/entrez/utils/fref.fcgi?http://amigo.geneontology.org/cgi-bin/amigo/go.cgi?view=details&depth=1&query=6512) ([ISG15-specific protease activity](http://www.ncbi.nlm.nih.gov/entrez/utils/fref.fcgi?http://amigo.geneontology.org/cgi-bin/amigo/go.cgi?view=details&depth=1&query=19785)) | Usp18 | ubiquitin specific peptidase 18 | NM_011909 | 2.629+/-0.762 |
| [defense response](http://www.ncbi.nlm.nih.gov/entrez/utils/fref.fcgi?http://amigo.geneontology.org/cgi-bin/amigo/go.cgi?view=details&depth=1&query=6952) ([GPI anchor binding](http://www.ncbi.nlm.nih.gov/entrez/utils/fref.fcgi?http://amigo.geneontology.org/cgi-bin/amigo/go.cgi?view=details&depth=1&query=48503)) | Ly6c | lymphocyte antigen 6 complex, locus C | D86232 | 2.530+/-0.515 |
| [metabolic process](http://www.ncbi.nlm.nih.gov/entrez/utils/fref.fcgi?http://amigo.geneontology.org/cgi-bin/amigo/go.cgi?view=details&depth=1&query=8152) ([glutathione transferase activity](http://www.ncbi.nlm.nih.gov/entrez/utils/fref.fcgi?http://amigo.geneontology.org/cgi-bin/amigo/go.cgi?view=details&depth=1&query=4364)) | Gstm2 | glutathione S-transferase, mu 2 | NM_008183 | 2.21+/-0.586 |
| [methionine biosynthetic process](http://www.ncbi.nlm.nih.gov/entrez/utils/fref.fcgi?http://amigo.geneontology.org/cgi-bin/amigo/go.cgi?view=details&depth=1&query=9086) ([homocysteine S-methyltransferase activity](http://www.ncbi.nlm.nih.gov/entrez/utils/fref.fcgi?http://amigo.geneontology.org/cgi-bin/amigo/go.cgi?view=details&depth=1&query=8898)) | Bhmt | betaine-homocysteine methyltransferase | NM_016668 | 1.926+/-0.430 |
| RIKEN cDNA 8430408G22  fat-specific expressed | 8430408G22Rik | RIKEN cDNA 8430408G22 gene | NM_145980 | 1.931+/-0.678 |
| [retinol metabolic process](http://www.ncbi.nlm.nih.gov/entrez/utils/fref.fcgi?http://amigo.geneontology.org/cgi-bin/amigo/go.cgi?view=details&depth=1&query=42572) | Rbp1 | retinol binding protein 1, cellular | NM_011254 | 1.806+/-0.384 |
| [cell adhesion](http://www.ncbi.nlm.nih.gov/entrez/utils/fref.fcgi?http://amigo.geneontology.org/cgi-bin/amigo/go.cgi?view=details&depth=1&query=7155) ([extracellular matrix structural constituent](http://www.ncbi.nlm.nih.gov/entrez/utils/fref.fcgi?http://amigo.geneontology.org/cgi-bin/amigo/go.cgi?view=details&depth=1&query=5201)) | Col3a1 | collagen, type III, alpha 1 | NM_009930 | 1.785+/-0.259 |
| [defense response](http://www.ncbi.nlm.nih.gov/entrez/utils/fref.fcgi?http://amigo.geneontology.org/cgi-bin/amigo/go.cgi?view=details&depth=1&query=6952) ([GPI anchor binding](http://www.ncbi.nlm.nih.gov/entrez/utils/fref.fcgi?http://amigo.geneontology.org/cgi-bin/amigo/go.cgi?view=details&depth=1&query=48503)) | Ly6e | lymphocyte antigen 6 complex, locus E | NM_008529 | 1.767+/-0.345 |
| [cell-cell signaling](http://www.ncbi.nlm.nih.gov/entrez/utils/fref.fcgi?http://amigo.geneontology.org/cgi-bin/amigo/go.cgi?view=details&depth=1&query=7267) (ISG15-protein conjugation) | G1p2 | interferon, alpha-inducible protein | NM_015783 | 1.765+/-0.656 |
| [apoptosis](http://www.ncbi.nlm.nih.gov/entrez/utils/fref.fcgi?http://amigo.geneontology.org/cgi-bin/amigo/go.cgi?view=details&depth=1&query=6915) | Bbc3 | Bcl-2 binding component 3 | NM_133234 | 1.748+/-0.476 |
| [angiogenesis](http://www.ncbi.nlm.nih.gov/entrez/utils/fref.fcgi?http://amigo.geneontology.org/cgi-bin/amigo/go.cgi?view=details&depth=1&query=1525) ([cytoskeletal protein binding](http://www.ncbi.nlm.nih.gov/entrez/utils/fref.fcgi?http://amigo.geneontology.org/cgi-bin/amigo/go.cgi?view=details&depth=1&query=8092)) | Anxa2 | annexin A2 | D10024 | 1.643+/-0.648 |
| [GPI anchor binding](http://www.ncbi.nlm.nih.gov/entrez/utils/fref.fcgi?http://amigo.geneontology.org/cgi-bin/amigo/go.cgi?view=details&depth=1&query=48503) (bone marrow stromal cell antigen 2) | Bst2 | bone marrow stromal cell antigen 2 | NM_198095 | 1.612+/-0.331 |
| [protein binding](http://www.ncbi.nlm.nih.gov/entrez/utils/fref.fcgi?http://amigo.geneontology.org/cgi-bin/amigo/go.cgi?view=details&depth=1&query=5515) | A930016D02Rik | leucine-rich repeats and transmembrane domains 1 | NM_176920 | 1.591+/-0.364 |
| [galactose binding](http://www.ncbi.nlm.nih.gov/entrez/utils/fref.fcgi?http://amigo.geneontology.org/cgi-bin/amigo/go.cgi?view=details&depth=1&query=5534) | Lgals1 | lectin, galactose binding, soluble 1 | NM_008495 | 1.555+/-0.662 |
| [blood coagulation](http://www.ncbi.nlm.nih.gov/entrez/utils/fref.fcgi?http://amigo.geneontology.org/cgi-bin/amigo/go.cgi?view=details&depth=1&query=7596) ([calcium ion binding](http://www.ncbi.nlm.nih.gov/entrez/utils/fref.fcgi?http://amigo.geneontology.org/cgi-bin/amigo/go.cgi?view=details&depth=1&query=5509)) | Anxa5 | annexin A5 | NM_009673 | 1.52+/-0.509 |
| [organic anion transport](http://www.ncbi.nlm.nih.gov/entrez/utils/fref.fcgi?http://amigo.geneontology.org/cgi-bin/amigo/go.cgi?view=details&depth=1&query=15711) | Slc22a7 | solute carrier family 22 (organic anion transporter), member 7 | NM_144856 | 1.415+/-0.225 |
| [transport](http://www.ncbi.nlm.nih.gov/entrez/utils/fref.fcgi?http://amigo.geneontology.org/cgi-bin/amigo/go.cgi?view=details&depth=1&query=6810) ([ammonium transporter activity](http://www.ncbi.nlm.nih.gov/entrez/utils/fref.fcgi?http://amigo.geneontology.org/cgi-bin/amigo/go.cgi?view=details&depth=1&query=8519)) | Rhbg | Rhesus blood group-associated B glycoprotein | NM_021375 | 1.373+/-0.200 |
| [negative regulation of cell proliferation](http://www.ncbi.nlm.nih.gov/entrez/utils/fref.fcgi?http://amigo.geneontology.org/cgi-bin/amigo/go.cgi?view=details&depth=1&query=8285) | Ifitm3 | interferon induced transmembrane protein 3 | NM_025378 | 1.34+/-0.256 |
| Lipid metabolism * ([hydroxymethylglutaryl-CoA synthase activity](http://www.ncbi.nlm.nih.gov/entrez/utils/fref.fcgi?http://amigo.geneontology.org/cgi-bin/amigo/go.cgi?view=details&depth=1&query=4421)) | HMGCs1 | 3-hydroxy-3-methylglutaryl-Coenzyme A synthase 1 | NM_145942 | 1.325+/-0.334 |
| [cholesterol biosynthetic* process](http://www.ncbi.nlm.nih.gov/entrez/utils/fref.fcgi?http://amigo.geneontology.org/cgi-bin/amigo/go.cgi?view=details&depth=1&query=6695) ([7-dehydrocholesterol reductase activity](http://www.ncbi.nlm.nih.gov/entrez/utils/fref.fcgi?http://amigo.geneontology.org/cgi-bin/amigo/go.cgi?view=details&depth=1&query=47598)) | Dhcr7 | 7-dehydrocholesterol reductase | NM_007856 | 1.283+/-0.287 |
| [acute-phase response](http://www.ncbi.nlm.nih.gov/entrez/utils/fref.fcgi?http://amigo.geneontology.org/cgi-bin/amigo/go.cgi?view=details&depth=1&query=6953) ([lipid transporter activity](http://www.ncbi.nlm.nih.gov/entrez/utils/fref.fcgi?http://amigo.geneontology.org/cgi-bin/amigo/go.cgi?view=details&depth=1&query=5319)*) | Saa1 | serum amyloid A1 | NM_009117 | 1.192+/-0.471 |
| [acute-phase response](http://www.ncbi.nlm.nih.gov/entrez/utils/fref.fcgi?http://amigo.geneontology.org/cgi-bin/amigo/go.cgi?view=details&depth=1&query=6953) ([lipid transporter activity](http://www.ncbi.nlm.nih.gov/entrez/utils/fref.fcgi?http://amigo.geneontology.org/cgi-bin/amigo/go.cgi?view=details&depth=1&query=5319)*) | Saa3 | serum amyloid A 3 | NM_011315 | 1.156+/-0.471 |
| [zinc ion homeostasis](http://www.ncbi.nlm.nih.gov/entrez/utils/fref.fcgi?http://amigo.geneontology.org/cgi-bin/amigo/go.cgi?view=details&depth=1&query=6882) (zinc transporter) | Slc39a4 | solute carrier family 39 (zinc transporter), member 4 | NM_028064 | 1.115+/-0.224 |
| [zinc ion binding](http://www.ncbi.nlm.nih.gov/entrez/utils/fref.fcgi?http://amigo.geneontology.org/cgi-bin/amigo/go.cgi?view=details&depth=1&query=8270) (zinc finger, FYVE domain containing 21 ) | Zfyve21 | zinc finger, FYVE domain containing 21 | NM_026752 | 1.090+/-0.212 |
| [defense response](http://www.ncbi.nlm.nih.gov/entrez/utils/fref.fcgi?http://amigo.geneontology.org/cgi-bin/amigo/go.cgi?view=details&depth=1&query=6952) ([N-methyltransferase activity](http://www.ncbi.nlm.nih.gov/entrez/utils/fref.fcgi?http://amigo.geneontology.org/cgi-bin/amigo/go.cgi?view=details&depth=1&query=8170)) | Hrmt1l2 | heterogeneous nuclear ribonucleoproteins methyltransferase-like 2 | AK077046 | 1.01+/-0.158 |
